# Volumetric measurement of dentoalveolar defects by means of intraoral 3D scanner and gravimetric model

**DOI:** 10.1007/s10266-018-00410-6

**Published:** 2019-01-07

**Authors:** Martin J. R. Lindström, Marianne Ahmad, Ryo Jimbo, Arman Ameri, Per Vult Von Steyern, Jonas P. Becktor

**Affiliations:** 1 Department of Oral and Maxillofacial Surgery and Oral Medicine, Faculty of Odontology, Malmö University, Carl Gustafs Väg 34, 214 21 Malmö, Sweden; 2 Department of Prosthodontics, Faculty of Odontology, Malmö University, Malmö, Sweden; 3 Department of Materials Science and Technology, Faculty of Odontology, Malmö University, Malmö, Sweden

**Keywords:** Surgical techniques, Wound healing, Volumetric measurement, Tissue alterations, Intraoral scanning

## Abstract

The aim of this study was to evaluate the accuracy in volumetric measurements obtained on an experimental model using an intraoral scanner and a gravimetric method. Three identical partial dentate maxillary acrylic models with three fabricated alveolar defects, in anterior and posterior regions, were scanned using an intraoral scanner (20 scans/defects). The defects differed in terms of size and distance of neighbouring teeth. As references, replicas of each defect were created using a dimensional stable silicone impression material. After measuring the mass of each replica, the volume was calculated by dividing the mass of each replica by the density of the impression material. The defects had a volume, according to the gravimetric method, ranging from 40.5 to 143.7 mm^3^. The scans were imported to metrology software for analyses. Accuracy was determined in terms of trueness and precision. The mean trueness for all defect types was 0.168 mm^3^ (SD 0.691, range 2.82). There was no statistical significant difference between the mean trueness for all defects measured (*p* = 0.910). The mean precision for all defect types was 0.147 mm^3^ (SD 0.524, range 2.86). There were no statistical significant differences between the dental models in regard to mean precision (*p* = 0.401), however, there were statistical significant differences between defects in position 1 and 2 (*p* = 0.002) and 1 and 3 (*p* = 0.001). Based on the findings of this study, the intraoral scanner utilized in the current study presented an acceptable level of accuracy when measuring volume of defects.

## Introduction

Surgical interventions in oral and maxillofacial surgery often engage both soft and hard tissue and they often result in tissue alteration. The interventions may include all type of surgical traumas that generate physiological responses, such as extraction, tissue augmentation and implant treatment, important to understand in the prediction and evaluation of clinical treatment outcome. Bone remodelling is one of the central mechanisms behind the dimensional changes observed after surgical trauma, influencing volumetric dimensions of the bone tissue as well as the soft tissue topography. The ability to monitor and accurately measure volumetric changes in tissues is an important instrument of great value for the clinical evaluation and follow-up of different surgical procedures.

Various methods have been described for the measurement of soft and hard tissue alterations, including conventional X-rays [1], computer tomography (CT) [2], stereoscopic imaging [3], optical scanning [4–11], direct measurements intraorally or on casts [2, 12], or mapping by ultrasonic assessments [13, 14]. However, there are only limited data available regarding the accuracy of these techniques in vivo. Further, the techniques involving ionizing radiation may be questioned due to their invasiveness and the need for radiation exposure. These disadvantages may be compensated by the use of other techniques such as optical scanning and clinical photographs. However, a problem with techniques using images is the superimposing and matching in one common coordinate system, which is critical to enable precise measurements. In addition, the intraoral 3D scanner (IOS) itself has expected technology-related errors to a certain level, due to a large number of pieces of measured data that are linked together to profile a 3D object, resulting in distortion of the created data.

The quality of measurements is therefore defined by terms of accuracy. Accuracy itself is the combination of two elements, both important and complementary: “trueness” and “precision”. Trueness refers to the ability of a measurement to match the actual value of the object being measured, and that little or nothing deviates from reality. To detect the trueness from IOS, it is mandatory to have a reference model with error tending to zero. Although trueness is the key element for an IOS, it is not sufficient, as it must be accompanied by precision. Precision is defined as the ability of a measurement to be consistently repeated, ensuring that multiple measurements of the same object must necessarily be comparable and differ from each other as little as possible. To measure the precision of an IOS, no reference models are needed: it is sufficient to superimpose different intraoral scans, and evaluate to what extent they deviate, using a dedicated software.

In recent years, the development of IOS in the field of dentistry has resulted in numerous advancements. The IOS comprises the first component in the CAD/CAM system, which captures data of anatomical structures with the use of technologies such as confocal laser scanning, confocal microscopy, active triangulation, wavefront sampling or optical coherent tomography [15, 16].

Several studies have evaluated the accuracy of IOS [17–19]. Flügge et al. evaluated the precision of the iTero (Cadent), TRIOS (3Shape) and True Definition (3M ESPE) by the digitization of scanbodies. They found that the precision of the IOS decreased with an increasing distance between the scanbodies when compared to a dental lab scanner (D250, 3Shape) [17].

Previous studies by Müller et al. [20] investigated the impact of different scanning strategies of the TRIOS (3Shape) IOS. They demonstrated that the precision of the scans was lowered when the scanning started with covering the buccal surfaces of the teeth in a full dental arch. Contrary, there was a higher degree of precision when the scanning started with covering the palatal surfaces or in a back and forth manner [20]. These findings demonstrated that the scanning technique itself also seems to have an impact on the accuracy of the IOS.

The IOS system provides a level of accuracy that may be beneficial in analysing the alterations before and after surgical interventions. This technique would provide an additional, less invasive modality to evaluate the outcome of surgical procedures. However, to our knowledge, no previous studies have examined the accuracy of volumetric measurements using an IOS. In such setting, there is a need of a volume sensitive model to use as reference. In the field of physics, volume is commonly calculated by gravimetric analysis with very high accuracy [21]. Thus, it would be of significant interest to test the accuracy of a commercially available device to determine whether it can be utilized for such volumetric evaluation. Hence, the aim of this study was to evaluate the accuracy of an IOS in terms of volume measurements.

## Materials and methods

### Models

**Fig. 1:**
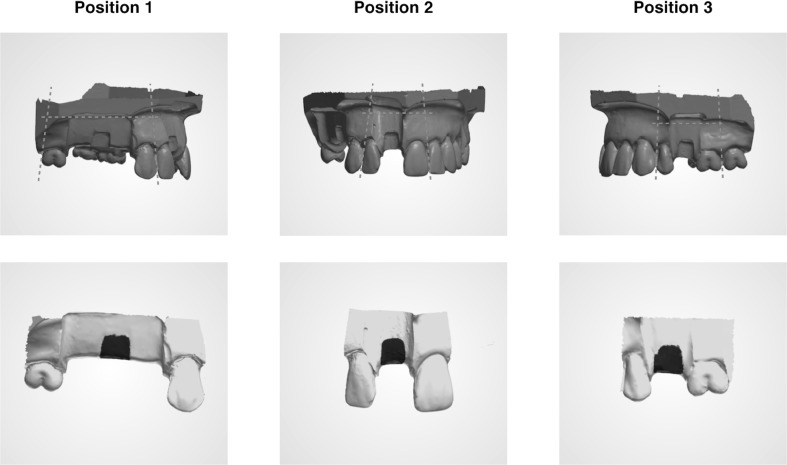
Aligned scans. 3D scan of acrylic model with alveolar defects in position 1 (tooth 15), position 2 (tooth 11) and position 3 (tooth 25). The volume of each defect was evaluated following superimposing and cropping using 3D processing software

**Fig. 2:**
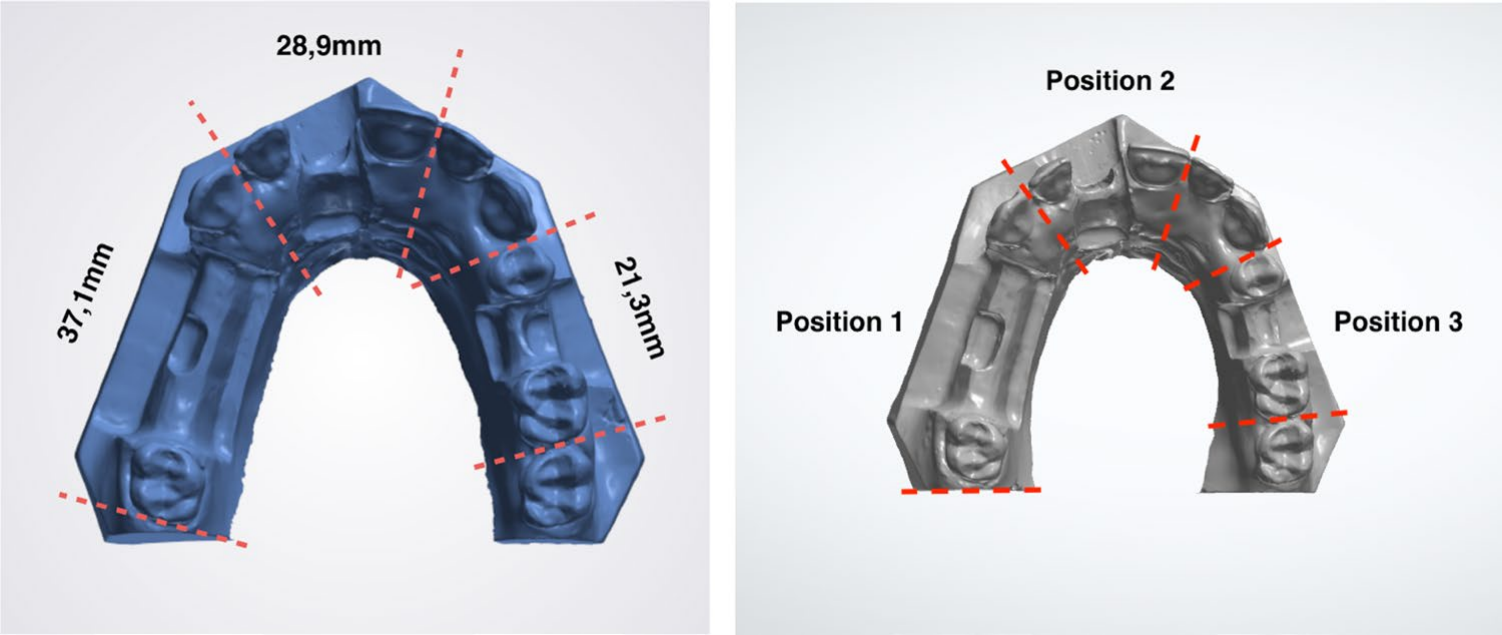
Acrylic model. The distance between neighbouring teeth in the acrylic model varied depending on the defect position

Three identical acrylic models of a partial dentate maxilla were used. The models were prepared identically with a defect on the alveolar ridge in the region of tooth number 15, 11 and 25 (referred to as position 1, 2 and 3) (Fig. 1). Each defect differed in size and distance between neighbouring teeth depending on the position of the defect, further presented in (Fig. 2).

### Intraoral scanner

The Trios 3^®^ Cart scanner used in this study is the third-generation IOS fabricated by 3-Shape (Copenhagen, Denmark). It works under the principle of confocal microscopy and optical scanning. Trios 3^®^ is a fast structured light scanner. It is powder-free and it produces in-colour images. The acquisition software of Trios 3^®^ produces proprietary files (.DCM) which can be exported to an open file format (.STL) file by the use of an export plugin provided by the manufacturer.

### Gravimetric method

The physical volume of the defects was determined by creating a replica of each defect using the silicone impression material Imprint™4 Bite (3M ESPE). The volume of the replica was quantified by the use of Archimedes’ principle, by weighing the impression and dividing the mass of each sample with the specific density of set Imprint™4 Bite [21].

Imprint™4 Bite was injected into each defect and left to set in accordance with the recommendations from the manufacturer. While setting, the impression material was contoured using a silicone key pre-fabricated in Putty™ (3M ESPE). Once set, the impression material was removed from the defect. Two impression samples were produced per defect. The samples were placed on a precision weighing balance with a readability of 0.001 g (Voyager^®^ Pro, Ohaus Corporation, Pine Brook, USA) and the average weight of the two impression samples was taken as the weight of the impression sample for that given defect. The average weight of each impression sample was divided by the known density (1654 gm/mL) for set Imprint ™4 Bite, which was established by the Technical Research Institute of Sweden. This calculation provided the reference volume for each of the defects.

### Study protocol

The test volumes were created by scanning of each defect with neighbouring teeth as reference points (one tooth on each side of the defect). Each defect was scanned 20 times by the IOS, following a previous session of learning scans, to avoid any inaccuracies due to learning curve. The scanning procedure of each defect was made with and without the replica. All the STL datasets from the IOS were loaded into 3D evaluation software Geomagic Qualify™ 2012 (Geomagic, Morrisville, USA). Each scan pair was aligned by superimposition in one common coordinate system using the best fit algorithm to generate the digital volume of the defects. To ensure a precise superimposition, unnecessary information were removed using the “cut with planes” function to trim the digital models as uniform as possible.

### Trueness and precision

For the trueness measurements, the digital datasets of the volume obtained from the scanner were compared to the physical reference volume from the replica of each defect.

For the precision measurements, the digital datasets of volume obtained from the scanner were compared in terms of deviation.

Only absolute values were used for the analysis, resulting in one value per two scans, in total 120 values.

### Statistical analyses

To detect differences regarding the trueness and precision of volume measurements, a univariate analysis of variance with a full factorial design was fitted. Further, Tukey test and post hoc analysis was implemented. The level of statistical significance was set to < 0.05. Least-square means were calculated with a 95% confidence interval. Box plots were used to illustrate experiment results. All calculations were performed using the statistical software SPSS Statistics (IBM Corp. IBM SPSS for Windows, Version 22.0. Armonk, NY: IBM Corp).

## Results

Mean values for trueness and precision are divided in regard to defect position. The univariate analysis with post hoc multiple comparison for observed means, revealed no statistical significant differences between models, stating there were no difference in regard to trueness nor precision dependent on defect size. However, statistical differences were observed regarding precision and defect position.

### Trueness

**Fig. 3:**
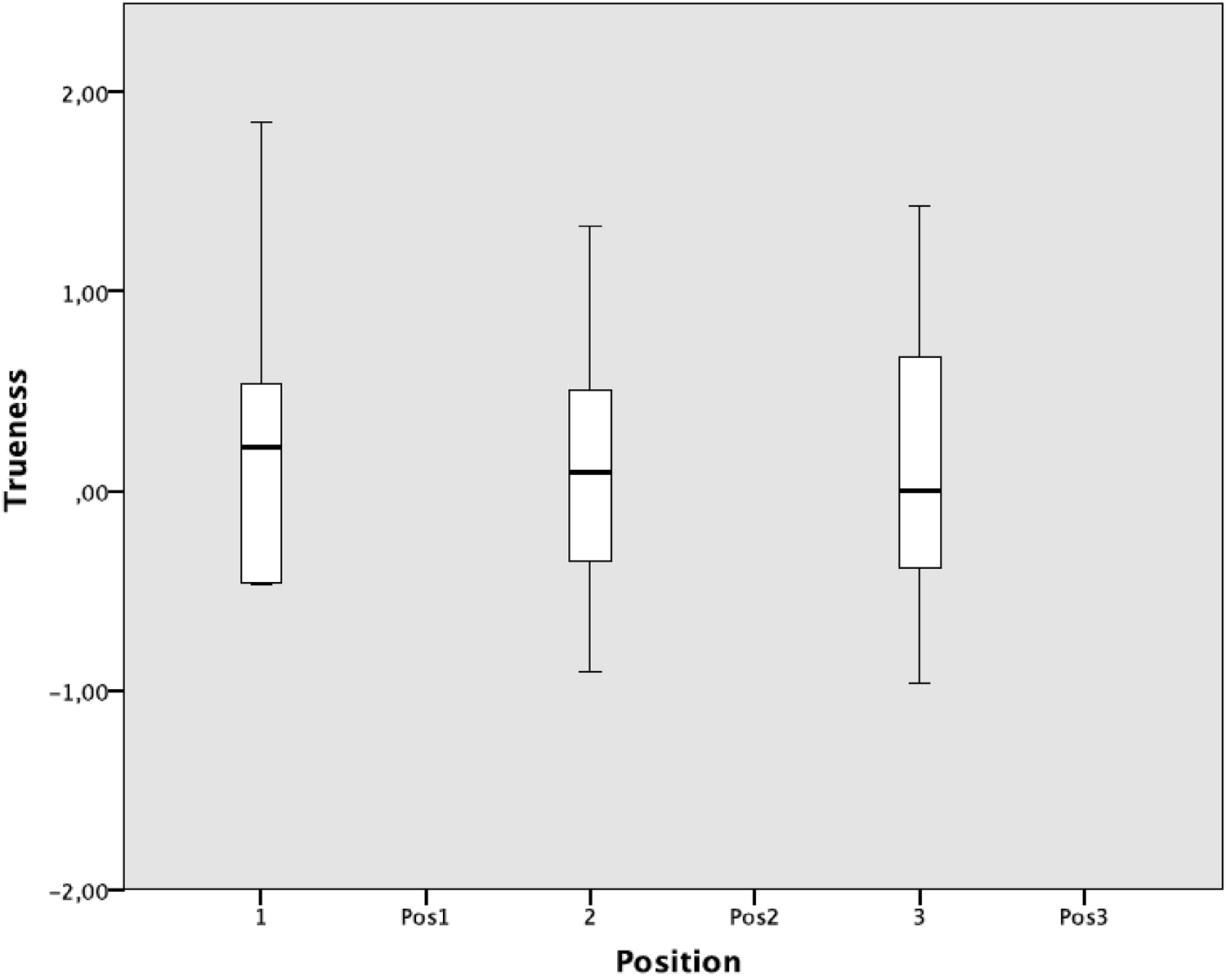
Trueness. Box plot of mean trueness of defects in positions 1, 2 and 3

The mean trueness was 0.168 mm^3^ (SD 0.691, range 2.82). For defects in position 1, the mean trueness was 0.236 mm^3^ (SD 0.708, range 2.32), position 2, 0.151 mm^3^ (SD 0.688, range 2.24) and position 3, 0.104 mm^3^ (SD 0.745, range 2.40) (Fig. 3). There was no statistical significant difference between the mean trueness for defects in positions 1, 2 or 3 (*p* = 0.910).

### Precision

**Fig. 4:**
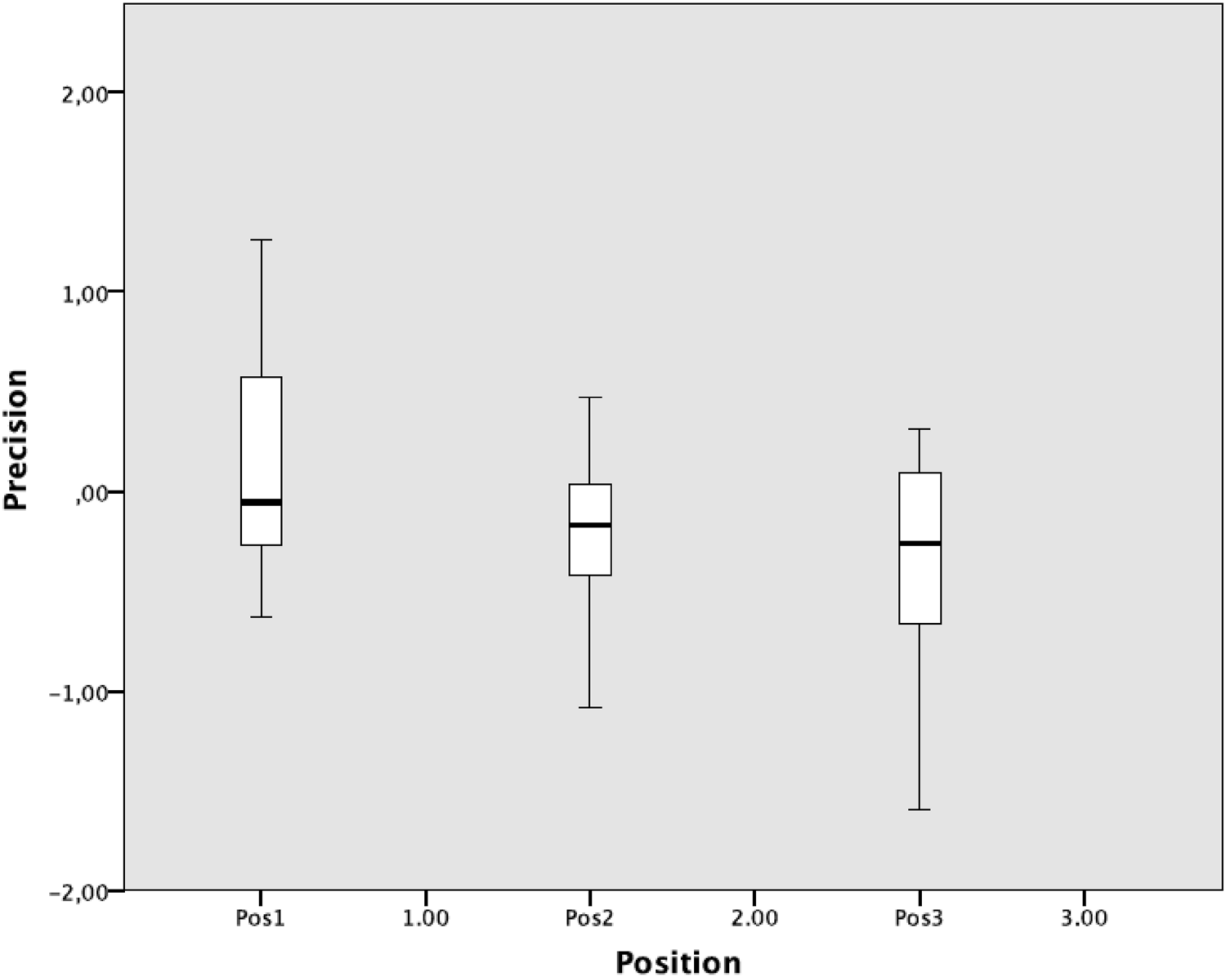
Precision. Box plot of mean precision of defects in positions 1, 2 and 3

The mean precision was 0.147 mm^3^ (SD 0.524, range 2.86). For defects in position 1, the mean precision was 0.118 mm^3^ (SD 0.525, range 1.89), in position 2, 0.202 mm^3^ (SD 0.391, range 1.55) and position 3, 0.356 mm^3^ (SD 0.542, range 1.91) (Fig. 4). There were no statistical significant differences between the different dental models, (*p* = 0.401). Analysis of deviations regarding mean differences dependent on defect position yielded statistical significant differences between defect positions 1 and 2 (*p* = 0.002), 1 and 3 (*p* = 0.001), but not between 2 and 3 (*p* = 0.200) The greatest deviation in mean difference was observed between defect positions 1 and 3, 0.473 mm3.

## Discussion

Volumetric analysis of oral tissues has evolved from rough visual inspection to more advanced techniques including cone-beam CT and recently the development of optical scanning technologies. CBCT is a frequently used modality with high accuracy in the surgical evaluation of hard tissue outcome. However, in the post-operative situation with an implant in vicinity of the region of interest, the measurement of data becomes complex due to metallic reflections [22]. The 3D optical scanning technique has mainly been applied to alter conventional dental impressions and facilitate the CAD/CAM process in prosthodontic treatment and orthodontic planning. But at a research level, the technique has fairly gained more attention as a method to provide volumetric measurements for evaluating soft and hard tissue alterations after surgical interventions [7, 23, 24]. Further abilities to assemble data from different types of 3D devices (IOS files and CT files) have further improved the so-called digital workflow [25], creating new possibilities such as treatment with guided implant surgery. The use of IOS for volumetric measurement is a simple and non-invasive tool that can provide 3D display making the volumetric analysis only limited to the accuracy of the IOS system.

The volumetric measurements of the present investigation suggested that the TRIOS intraoral scanner possessed high levels of accuracy. However, it was also demonstrated that there was a statistical significant difference between the positions of each defect in terms of precision. This is speculated to be mainly due to different anatomic morphologies and various scan area sizes. It has been reported that the individual patient anatomy is important to take into consideration prior to intraoral scanning. It can be said that depending on the site in the oral cavity, the degree of concavity and convexity can vary and the precision can be influenced. The influence of scan size has been reported by Flügge et al. [17] that an increased scanning area lowered the degree of precision using an intra-oral scanner [17, 18]. This may be the reason for the minor differences observed in the current study, however, it must also be stated that the observed difference might not have an impact in a clinical setting when comparing volumetric differences.

Several studies have utilized a 3D scanning technique to investigate the volume alterations following different surgical interventions [7, 10, 11, 23, 26–28]. Fickl et al. [7, 27] evaluated the dimensional changes in alveolar bone following tooth extractions in a dog mandible. In the studies from 2008, the volume difference was presented in millimetres following a 2D section of a 3D model, for instance, in a bucco/lingual section. In the latter study in 2009, the volume change was presented as the measured volume difference per measured area. Hence, the extent of volume change is not possible to compare with the defects in the present study. The change of volume alteration during soft tissue augmentation was analysed in a dog study with the help of a 3D scanning technique by Thoma et al. [8]. However, the volumetric changes were measured after soft tissue augmentation with different collagen-based matrices in the dog oral cavity, where they presented the volume change per measured area in millimetre. The presented outcome was the same as in two later studies, where they investigated the volume alteration of peri-implant tissues after tooth extraction by Schneider et al. [10, 11]. This did not reveal the total volume of each area of interest, which complicates any comparisons with regard to volume of defects used in the present study.

Gonzalez-Martin et al. [24] investigated the volume alteration following sub-epithelial connective tissue grafting. They used a soft tissue graft, which was harvested pre-prosthetically from the palate of each subject and placed in a sub-epithelial manner on the buccal side in patients lacking a central or lateral incisor. Conventional impressions were taken before and after the surgical treatment, which was latter quantified using a laboratory 3D scanner. They found a mean volume increase of 35.9mm^3^ with a range of 12.8–52.6 mm^3^ [24]. One can hypothesize that the deviations of volumetric measurements by the intraoral scanner in our study are still within what is a clinically acceptable level of inaccuracy.

Our present study has certain limitations. Foremost, it is an in vitro study. Therefore, the important results obtained here should be necessarily translated into the clinical setting, and validated in vivo, where there are additional factors that can degrade the quality of a scan (saliva, blood, accessibility to the surface of interest and movement of the patient).

Second, although the gravimetric method is a powerful method to calculate volume, it is highly dependent on the choice of material, its ability to flow and fill the volume of interest and its stability upon removal. The use of reference scanners such as optical desktop scanners or MicroCT are highly accurate alternative reference models for measurement of volume and surfaces. Although, it must be remembered that these instruments are limited to in vitro and ex vivo studies and thus are unavailable in a clinical setting. In conclusion, further studies at a clinical level are needed to confirm the outcomes of the present work, and to provide even more interesting data to clinicians.
